# Expectations and Requirements of Surgical Staff for an AI-Supported Clinical Decision Support System for Older Patients: Qualitative Study

**DOI:** 10.2196/57899

**Published:** 2024-12-17

**Authors:** Adriane Uihlein, Lisa Beissel, Anna Hanane Ajlani, Marcin Orzechowski, Christoph Leinert, Thomas Derya Kocar, Carlos Pankratz, Konrad Schuetze, Florian Gebhard, Florian Steger, Marina Liselotte Fotteler, Michael Denkinger

**Affiliations:** 1 Department for Orthopedic Trauma, Ulm University Medical Center, Ulm, Germany; 2 Institute for Geriatric Research, Ulm University Hospital, Zollernring 26, Ulm, 89073, Germany, 49 731 1870; 3 Department of Sociology, Institute of Sociology, Johannes Kepler University, Linz, Austria; 4 Institute of History, Philosophy and Ethics in Medicine, Ulm University, Ulm, Germany; 5 Agaplesion Bethesda Clinic Ulm, Ulm, Germany; 6 DigiHealth Institute, Neu-Ulm University of Applied Sciences, Neu-Ulm, Germany

**Keywords:** traumatology, orthogeriatrics, older adult, elderly, older people, aging, interviews, mHealth, mobile health, mobile application, digital health, digital technology, digital intervention, CDSS, clinical decision support system, artificial intelligence, AI, algorithm, predictive model, predictive analytics, predictive system, practical model, decision support, decision support tool

## Abstract

**Background:**

Geriatric comanagement has been shown to improve outcomes of older surgical inpatients. Furthermore, the choice of discharge location, that is, continuity of care, can have a fundamental impact on convalescence. These challenges and demands have led to the SURGE-Ahead project that aims to develop a clinical decision support system (CDSS) for geriatric comanagement in surgical clinics including a decision support for the best continuity of care option, supported by artificial intelligence (AI) algorithms.

**Objective:**

This qualitative study aims to explore the current challenges and demands in surgical geriatric patient care. Based on these challenges, the study explores the attitude of interviewees toward the introduction of an AI-supported CDSS (AI-CDSS) in geriatric patient care in surgery, focusing on technical and general wishes about an AI-CDSS, as well as ethical considerations.

**Methods:**

In this study, 15 personal interviews with physicians, nurses, physiotherapists, and social workers, employed in surgical departments at a university hospital in Southern Germany, were conducted in April 2022. Interviews were conducted in person, transcribed, and coded by 2 researchers (AU, LB) using content and thematic analysis. During the analysis, quotes were sorted into the main categories of geriatric patient care, use of an AI-CDSS, and ethical considerations by 2 authors (AU, LB). The main themes of the interviews were subsequently described in a narrative synthesis, citing key quotes.

**Results:**

In total, 399 quotes were extracted and categorized from the interviews. Most quotes could be assigned to the primary code *challenges in geriatric patient care* (111 quotes), with the most frequent subcode being *medical challenges* (45 quotes). More quotes were assigned to the primary code *chances of an AI-CDSS* (37 quotes), with its most frequent subcode being *holistic patient overview* (16 quotes), then to the primary code *limits of an AI-CDSS* (26 quotes). Regarding the primary code *technical wishes* (37 quotes), most quotes could be assigned to the subcode *intuitive usability* (15 quotes), followed by *mobile availability and easy access* (11 quotes). Regarding the main category *ethical aspects of an AI-CDSS*, most quotes could be assigned to the subcode *critical position* toward *trust in an AI-CDSS* (9 quotes), followed by the subcodes *respecting the patient’s will and individual situation* (8 quotes) and *responsibility remaining in the hands of humans* (7 quotes).

**Conclusions:**

Support regarding medical geriatric challenges and responsible handling of AI-based recommendations, as well as necessity for a holistic approach focused on usability, were the most important topics of health care professionals in surgery regarding development of an AI-CDSS for geriatric care. These findings, together with the wish to preserve the patient-caregiver relationship, will help set the focus for the ongoing development of AI-supported CDSS.

## Introduction

Older adults represent a large proportion of patients in hospitals, and numbers will be increasing [1]. Treatment of these patients can be challenging, due to complex medical conditions including deficits in multiple geriatric domains [2,3]. Therefore, identification of geriatric patients with complex needs is needed and should be followed by comprehensive geriatric assessments to capture needs and resources in multiple domains such as cognition, nutrition, physical functioning, comorbidities, frailty, and others [4].

An additional challenge is determining the best discharge destination for these patients, also referred to as transition of care or continuity of care (COC) [5]. Multiple options exist in most high-resource countries, such as rehabilitation with geriatric or orthopedic specialization, specialized acute geriatric departments or hospitals, short- or long-term care in nursing homes, or ideally a direct discharge home with or without further support [6]. In Germany, social services assist with organizing adequate COC. Ideally, the decision is discussed by physicians, patients, relatives, nurses, therapeutic, and social service staff. Barriers to optimal COC decisions have been observed and include staffing shortages, difficulties to assess the potential of geriatric patients in an acute stress situation, difficulties to communicate with relatives and team members in a structured way, and an insufficient alignment between bed capacity and the demand in the target facilities [5,7,8].

The involvement of geriatric specialists in the treatment of geriatric patients on surgical wards has been shown to improve relevant clinical outcomes [9]. However, a shortage of geriatric specialists prevents the widespread implementation of geriatric comanagement [10]. To address this problem, the SURGE-Ahead project (supporting surgery with geriatric comanagement and artificial intelligence [AI]) aims to develop an AI-enhanced clinical decision support system (AI-CDSS) to support geriatric comanagement and COC decisions in surgical departments without a resident geriatrician [11]. SURGE-Ahead started in July 2021 and is a multidisciplinary project funded by the Federal Ministry of Education and Research. For this purpose, the SURGE-Ahead AI-CDSS system collects patient data from the hospital information system (HIS) including central laboratory parameters, diagnoses, and operation procedures. In addition, relevant scores for comprehensive geriatric assessments domains, such as mobility, cognition including delirium, and medication appropriateness, will be registered, calculated, and presented to the surgical staff. Furthermore, a suggestion for the most suitable COC destination will be given based on decision trees and machine learning algorithms. To develop the algorithm, the ground truth (optimal discharge decision) will be provided by experts for geriatric medicine.

The use of an AI-CDSS in health care delivery raises ethical questions [12-14]. While it could improve communication with patients and contribute to increased quality of the diagnostic and therapeutic process [15,16], it also carries risks such as overlooking patients’ individual wishes and needs. Other issues, such as decreased trust in accuracy and usability of the system because of errors, technical problems, or overalerting, may result in the product not being used in everyday clinical practice [12,17]. It has been shown that a cocreation process with end users helps improve the development of targeted new digital health applications [18]. We therefore conducted qualitative expert interviews with different health care professionals to capture current challenges on surgical wards, requirements for an AI-CDSS, and ethical considerations. In detail, we aimed to cover the following 3 main categories:

1. Challenges and need for support in geriatric patient care.
2. Technical and general wishes regarding the development of an AI-CDSS for geriatric patient care in surgery.
3. Expected ethical challenges using an AI-CDSS with respect to the 4 core medico-ethical principles of autonomy, beneficence, maleficence, and justice [19].

## Methods

### Participants

We included 15 health care professionals involved in the inpatient surgical care of geriatric patients. Three senior physicians, 3 assistant physicians, and 3 nurses from the departments of urology, visceral surgery, and traumatology (all 3 departments are recruitment centers for the SURGE-Ahead project), as well as 2 senior physicians from the emergency department, 2 physiotherapists, and 2 social service workers, all responsible for surgical patients, agreed to participate. None of the interviewees were involved in the SURGE-Ahead project or in the development of the AI-CDSS.

Participants were recruited at Ulm University Medical Center in Southern Germany. The department of orthopedic trauma surgery has been certified as an AltersTraumaZentrum (center for geriatric traumatology) DGU in cooperation with a local geriatric clinic in 2019. This model of care entails medical visits by a geriatrician on trauma wards. Furthermore, a senior surgeon visits the geriatric clinic. Thus, an exchange exists between the 2 hospitals regarding the care for geriatric trauma patients. Currently, this model exists only for orthopedic trauma surgery and not for other surgical departments.

### Interviews

An interview guideline with 12 questions corresponding to the 3 main topics was prepared by a multidisciplinary research team consisting of geriatricians, surgeons, public health specialists, and ethicists. The questions were formulated on the basis of a prior literature research, the guidelines for conduct of qualitative interviews [20,21], and professional experience of the researchers. The number of questions was limited to 12 due to the expected short time available to our interview partners. Interviews were conducted in a semistructured way. While the preformulated questions set the focal point, ad hoc questions provided a possibility to clarify statements or focus on particularly important issues mentioned by the interviewees [22,23].

Interviews were conducted in person in April 2022 in German. Potential interview partners were contacted personally or via email. If willing to participate, interviewees received a short information sheet about the SURGE-Ahead project.

### Ethical Considerations

Ethical approval was waived by the ethics committee of Ulm University, because no information on personal data, health-related data, or data on sexuality of the interviewees was collected (March 21, 2022). All interviewees declared their consent after being informed about the aim of the study, their rights, potential risks, and data protection. Interviewees did not receive any compensation for participation in the study.

### Data Analysis

Interviews were recorded and transcribed by an external transcription service provider (abtipper.de). After transcription, the interviews were anonymized. Transcripts were checked for accuracy against the recordings by 2 authors (AU, LB) to avoid bias. Occurring flaws in transcription were resolved by mutual agreement.

The statements of the interviewees were coded, extracted, and sorted into main categories, primary codes, and subcodes according to usual procedures of qualitative analysis [24] and thematic analysis [25,26] using the software MAXQDA (VERBI GmbH). Total numbers of the extracted categories and codes are presented. Representative quotes that illustrate various themes were translated from German into English by 2 researchers independently (AU, MLF), compared, and then unified into a final version. Based on these representative quotes, a narrative synthesis of the results was written. Finally responses across categories were also stratified according to the most significant hopes and fears of participants [27]. The final manuscript was written based on COREQ (Consolidated Criteria for Reporting Qualitative Research) [28].

## Results

### Interview Participants

In total, 14 interviews were conducted with 15 professionals. The mean duration of the conducted interviews was 13:51 minutes. The interviewees’ professional experience ranged from 2 to 43 years with a median duration of 15 years. Median time of affiliation with the department (8 years) differed between professions. Nursing management had the longest affiliation of 22 years, and senior physicians in the emergency department had the shortest median affiliation of 1.5 years. For details, see Table 1.

**Table 1.** Interviewed experts^a^.

ID	Profession	Professional experience (years)	Department	Affiliation with the department (years)
1	Social service	32	Social counseling service	2
2	Social service	8	Social counseling service	8
3	Assistant physician	4	Visceral surgery	1
4	Nursing management	43	Urology	40
5	Senior physician	19	Traumatology	19
6	Physiotherapy	22	Physiotherapy surgery	22
7	Senior physician	21	ED^b^	1
8	Physiotherapy	34	Physiotherapy surgery	34
9	Nursing management	37	Traumatology	22
10	Assistant physician	2	Urology	2
11	Senior physician	15	ED	2
12	Assistant physician	6	Traumatology	6
13	Nursing management	8	Visceral surgery	8
14	Senior physician	10	Visceral surgery	9
15	Senior physician	13	Urology	13

a Identification numbers (ID) are assigned to the interview partners according to the chronological order of the interviews. In the narrative synthesis of the statements below, these IDs are assigned to the corresponding quotes.

b ED: emergency department.

### Qualitative Analysis of Interviews

#### Overview of Themes

Interview data were categorized into three major themes: (1) geriatric patient care, (2) use of an AI-CDSS in geriatric care, and (3) ethical challenges of using an AI-CDSS in geriatric care. Table 2 gives an overview of all extracted main categories (1-3), primary codes, and subcodes. A narrative synthesis of interview statements corresponding with the primary codes and their subcodes is represented thereafter. Key quotes are integrated into the text and additional quotes are listed in Multimedia Appendix 1 (selected quotes for each category).

**Table 2.** Main categories (1-3), primary codes, and subcodes emerging from inductive analysis of the interviews.

Main topic, primary codes, and subcodes			Number of quotes
1. Geriatric care			
	Challenges in geriatric patient care		111^a^
		Medical challenge	45
		Discharge management	27
		High workload	20
		Communication	14
		Lack of technical solutions	6
	Solutions for challenges in geriatric patient care		60^a^
		Patient-centered care	22
		Communication (patients, colleagues, and relatives)	21
		Social service	11
		Cooperation with geriatric hospital	6
	Wishes for geriatric patient care		20^a^
		Geriatric expertise	10
		Improved communication and availability of information	7
		Human resources and assistive devices	3
2. Use of an AI-CDSS^b^ in geriatric care			
	General wishes for an AI-CDSS^c^		53^a^
		Electronic health record	30
		Work support	22
		No added workload	1
	Chances of an AI-CDSS^c^		37^a^
		Holistic patient overview	16
		Improvement of geriatric patient care	13
		Acceleration of processes	5
		Decision support	3
	Technical wishes for an AI-CDSS		37^a^
		Intuitive usability	15
		Mobile availability and easy access	11
		Automatic transfer of HIS^d^ data into AI-CDSS	8
		Other	3
	Limits of an AI-CDSS		26^a^
		Decision-making authority remains with humans	9
		Preservation of individual and personal patient care	9
		Technical affinity and time constraints	7
		Information overload	1
3. Ethical aspects of an AI-CDSS in geriatric care			
	Critical assessment of AI-CDSS recommendations		23^a^
		Respecting the patient’s will and individual situation	8
		Beneficence	5
		Data protection	4
		Scientific validation	3
		Loss of professional autonomy	2
		Legal responsibility	1
	Trust in an AI-CDSS		15^a^
		Critical position	9
		Ambivalent position	4
		Rejection	2
	Preserving the patient-caregiver relationship		17^a^
		Responsibility remains in the hands of humans	7
		Inform patients about the use of an AI-CDSS	4
		Preservation of patient-caregiver relationship	3
		Use AI-CDSS without informing patients	3

a The total sum of quotes belonging to the subcodes.

b AI-CDSS: artificial intelligence–supported clinical decision support system.

c In the following section, wishes for and chances of an AI-CDSS are presented together due to their overlapping subcodes.

d HIS: hospital information system.

#### Geriatric Care

##### Challenges

**Figure 1.**
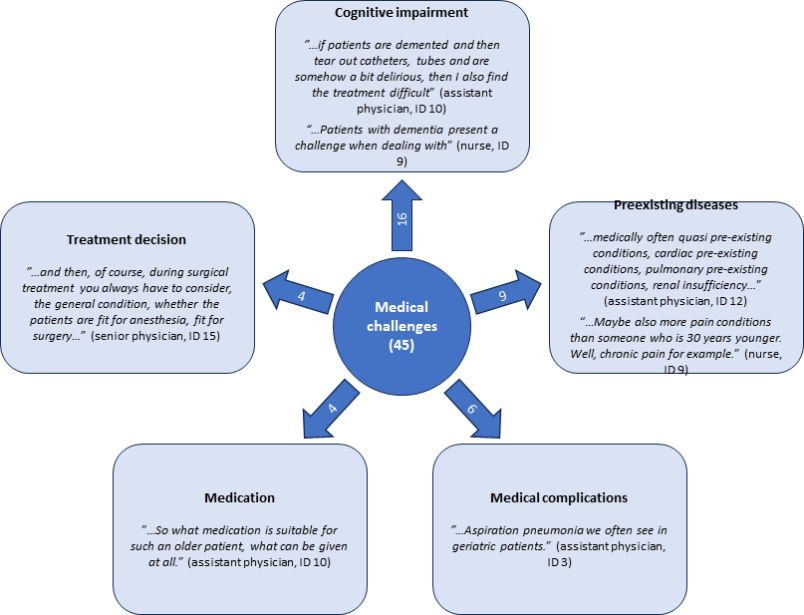
Numbers in the inner circle and the outgoing arrows show the number of citations for each subcode and the primary code. Further mentioned challenges were drainages (3 citations), reduced mobility (3 citations), slower regeneration (2 citations), and legal support (1 citation).

Physicians, nurses, and physiotherapists mentioned multiple medical challenges when caring for geriatric patients on surgical wards such as medical complications, preexisting diseases, and cognitive impairment. Figure 1 shows this primary code and its associated subcodes including key quotes.

In addition to medical challenges, discharge management was stressed especially by social service members as highly challenging in clinical routine due to the need to organize the best possible follow-up care for each geriatric patient (Multimedia Appendix 1, quote 1), alongside a high patient turnover and the resulting high workload in acute surgical hospitals. Social service members also stated the lack of a person having the main responsibility for the COC decision as an issue.

*…each day we have on average 25 to over 30 registrations. And you have to manage that for a start. And that is what is actually almost not possible.*

[Social service member, ID 1]

Physicians mentioned that they have difficulties to find the best place for the patient after discharge, because they do not feel properly educated about it (Multimedia Appendix 1, quote 2). Physicians, nurse management, and social service mentioned that staff shortage and high work density can impact the quality of care, which can be aggravated by economic pressure (Multimedia Appendix 1, quotes 3 and 4). Finally, interviewees still relied on telephone, fax, and email as the only technical solutions facilitating geriatric patient care and especially discharge management (Table S1 in Multimedia Appendix 1, quotes 5 and 6).

##### Solutions

Patient-centered care involving patient contact and diagnostic examinations, for example, in radiology or the laboratory, as well as exchanging ideas with colleagues and experts, are commonly cited strategies in geriatric patient care. While physicians, nurses, and physiotherapists see the social service as a crucial partner for organizing discharge of patients, social service members stated that they find value in involving relatives and having conversations with them. Interviewees also expressed that cooperation with the geriatric hospital is an important aspect in geriatric patient care on surgical wards.

*…, so you try to find a solution with the social service or, depending on the illness, together with ambulant palliative care (Brückenpflege), together with the relatives to find a solution. Bethesda (acute geriatric hospital) is of course also always a welcome solution.*

[Nurse ID 4]

##### Wishes

The main wish expressed by physicians, nurse management, and social service was an improved cooperation with geriatric experts.

*…perhaps if we had…people here who are more familiar with geriatric disease patterns. After all, we all rather come from the acute care, acute hospital. We all don’t really have a background in geriatric care, there are other basics involved in our training. So, if you could mix it up a little, it would probably be good for everyone, yes*.

[Nurse ID 13]

Nurses, physicians, and social service employees stressed the need for better communication, also across institutions, and timely availability of information (Multimedia Appendix 1, quotes 7 and 8). Further frequent wishes to improve geriatric patient care on surgical wards were improved awareness about drug interactions and side effects especially regarding geriatric patients (assistant physician) and improved management of delirium (senior and assistant physicians). More frequent mobilization of patients through physiotherapy (assistant physicians) and the establishment of a geriatric ward in the department for trauma surgery (senior physician) were mentioned by singular interviewees.

### Use of an AI-CDSS

#### General Wishes for and Chances of an AI-CDSS

All interviewees expressed a desire for an electronic health record (EHR) to gain a holistic picture of the patient’s situation, accelerate processes, and reduce resource expenses.

*…so I see a chance to shorten my time expenditure because I already have a medical history that I can access with a few clicks. Which might otherwise cost me a lot of time*.

[Social service ID 2]

Cognition, frailty, mobility, medication, substance abuse, social history, and laboratory values were mentioned as important aspects, which should be integrated in an AI-CDSS (Multimedia Appendix 1, quote 9). Primarily requested by physicians were features that could increase awareness for needs and pitfalls of geriatric patient care but could also give constructive solutions, for example, for drug interactions, delirium management, or discharge management (Multimedia Appendix 1, quotes 10 and 11). The capacity of the AI-CDSS to advance treatment effectiveness through faster evaluation of large amounts of data was mentioned (Multimedia Appendix 1, quote 12). Finally, the application should not increase the existing workload (Multimedia Appendix 1, quote 13).

#### Technical Wishes for an AI-CDSS

Intuitive usability was mentioned most frequently by all interviewees when asked about technical wishes for an AI-CDSS.

*So I think it should be intuitive. That’s always difficult to define. So it should be a clear program that I can access from anywhere. Maybe not only from fixed PCs, but also from tablets and so on…But it should be kept relatively simple so that everyone can understand it. And can use it without much prior knowledge*.

[Assistant physician ID 12]

Mobile and easy access for all involved caregivers were additional desirable features (Multimedia Appendix 1, quote 14). Physicians emphasized the importance of a regular and automatic data transfer from the HIS to the new AI-CDSS to avoid redundancy and additional workload due to the need for double entries (Multimedia Appendix 1, quotes 15 and 16). Some physicians also proposed linking the application with external hospitals for better networking and some suggested incorporating functions such as showing the availability of free beds, for example, in external rehabilitation hospitals (Multimedia Appendix 1, quote 17).

#### Limits of an AI-CDSS

The main concern expressed by interviewees regarding the use of an AI-CDSS was that the final decision-making authority should always stay with humans. They emphasized that the doctor-patient relationship cannot be replaced by a computer. In addition, some interviewees worried about losing sight of the individual patient’s needs when relying on an AI-CDSS:

*Limitations of course, it is and will remain a human being and not a material in quotation marks. That one does not forget this and that this is still considered and not forgotten, that there is no text-book and no cookie-cutter approach….*

[Assistant physician ID 12]

Several interviewees also raised concerns about the implementation of new technology in health care settings, particularly regarding the handling and operation of such systems. One interviewee mentioned that operating an AI-CDSS might be challenging in a busy clinical environment due to the already existing high workload (Multimedia Appendix 1, quotes 18 and 19). One senior physician mentioned that it might be difficult to make decisions about interventions, such as new medications, due to the theoretical risks and complications presented by the system (Multimedia Appendix 1, quote 20). Interestingly, 2 assistant doctors did not see any risks in incorporating such a new technology.

### Ethical Aspects of an AI-CDSS

#### Critical Assessment of AI-CDSS Recommendations

Many interviewees stated that they had no concerns about—and would even appreciate—the use of an AI-CDSS in geriatric patient care as a supporting tool. However, an underlying condition should be informing patients about the use of a CDSS and considering the patient’s individual situation. Interviewees stressed that the will of patients or relatives should be at the core of clinical decisions and COC decisions.

*So, the patient’s wishes should be considered. Or, if the patient can no longer decide for themselves, the relatives in any case.*

[Nursing ID 9]

In this regard, interviewees mentioned that it might be difficult for an AI-CDSS to capture all facets of geriatric patients (Multimedia Appendix 1, quote 21). One of the respondents described it as “*putting patients in a box*” (physiotherapist, ID 8).

According to the respondents, such a system would require solid scientific validation prior to integration into routine care and adhere to all data protection requirements (Multimedia Appendix 1, quote 22 and 23). Some concerns of the interviewees also raised the question of accountability and reliance on the decisions made by an AI-CDSS. Strong reliance on the technology might lead to a loss of autonomy and professional identity of caregivers. Moreover, the question of professional and legal responsibility for the decisions taken was raised (Multimedia Appendix 1, quote 24).

#### Trust in an AI-CDSS

The majority of interviewees were unsure whether they would fully trust the AI-CDSS and emphasized the need for medical professionals to critically evaluate all suggestions made by the system.

*So, I can imagine that it could well be trustworthy and reliable. However, I would not rely on that alone. So would say, it is…a recommendation. So not a strict default. So I think you still have to examine and evaluate the individual case from a human or medical point of view to see whether it really makes sense and whether it is in the best interests of the patient or their relatives. But as a supportive tool, I think it’s very good. And then it would also be reliable for me.*

[Senior doctor ID 5]

#### Preserving a Patient-Caregiver Relationship

Regarding the patient-caregiver relationship, most interviewees remarked that it is crucial to openly communicate the use of an AI-CDSS (Multimedia Appendix 1, quote 25) and to emphasize that the main responsibility for clinical decisions remains in the hands of humans. Preservation of the physical and emotional relation between caregivers and patients was also mentioned by interviewees as an important factor for a trusting relation.

*…by always clarifying, that the final medical decision always lies with a person or a doctor. I think that is very, very important for the patient, but also for the doctor himself…So I think, the independent decision for medical matters must remain with the doctor. And of course you can’t replace the doctor-patient-relationship with a computer, so to speak.…So it must always be clear that the doctor ultimately makes the decision, so to speak, and the machine supports him in making the right one. I would say that the machine processes a complexity of information that the doctor would otherwise not be able to process*.

[Senior doctor ID 7]

In contrast, 1 interviewee feared an adverse result for the patient-caregiver relationship if openly communicating about the use of an AI-CDSS and another interviewee proposed not to inform patients about the use of it (Multimedia Appendix 1, quote 26).

#### Fears and Hopes

**Figure 2.**
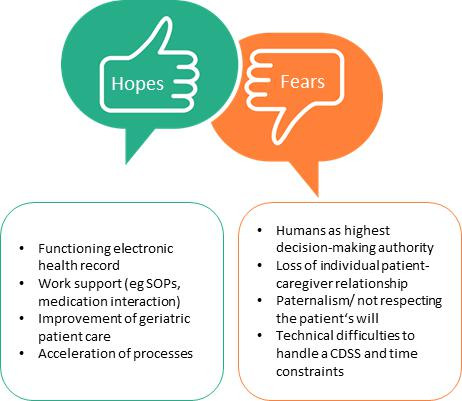
Main hopes and fears expressed by interviewees about an AI-CDSS for surgical patient care. CDSS: clinical decision support system; SOP: standard operating procedure.

In order to summarize all answers and for a quick and clear overview, responses were stratified according to the biggest fears and hopes that could be extracted across all categories and codes as displayed in Figure 2.

## Discussion

### Study Overview

In this qualitative study, we analyzed the status of geriatric care and the expectations for an AI-CDSS supporting patient care by interviewing physicians, nurses, physiotherapists, and social service members. Results were summarized in 3 main categories covering geriatric patient care, the use of an AI-CDSS, and ethical considerations regarding an AI-CDSS. While the biggest hope was that such an application could improve geriatric care including the handling of medical geriatric challenges within a functioning digital service, the loss of individual patient-caregiver relation and a lack of usability were the biggest fears.

### AI-CDSS to Support Geriatric Care

Provision of an EHR by an AI-CDSS was the most frequent subcode extracted from the interviews. Currently, health professionals must gather information about patients from different sources (eg, HIS, paper documents, and telephone calls), which is time consuming. One of the biggest advantages of a CDSS could be an overview of relevant individual clinical and social information. However, a higher workload was feared due to the task of entering data. Physicians already spend up to 35% of their time on documentation [29,30]. Because of these shortcomings low acceptance of EHR and CDSS is a common phenomenon in clinical practice [31,32]. Therefore, the involvement of future users in the development of the application [32], ensuring seamless integration with existing clinical information systems, minimizing the need for manual data entry, and designing applications as close as possible to the existing systems have been found to be important for the acceptance of CDSS [17,33].

Interviewees also emphasized how challenging it is to care for geriatric patients due to complex medical conditions and impairments such as cognitive impairment. It has been shown that a multidisciplinary approach is favorable for geriatric patient care and COC decisions [34-37]. Considering the lack of geriatricians [10], geriatric expertise is something the AI-CDSS could provide, and support clinical work, by highlighting relevant issues such as medication interactions or factors increasing the risk for delirium.

The lack of knowledge of optimal COC was considered relevant by all professionals. The discharge destination can have a profound effect on long-term outcomes. A discharge to nursing homes has been associated with lower survival than a discharge home [5]. COC placement suggestions by an AI-CDSS could fill a gap in care for geriatric patients in surgery. Some interviewees even expressed the wish that an AI-CDSS should enable reservation of beds in rehabilitation clinics. For this purpose, new software options for hospitals have already been established and are currently being implemented in several regions in Europe [38].

### Organizational and Technical Challenges of an AI-CDSS

Time and resource constraints could inhibit overall feasibility and practicality. Some interviewees were concerned whether all health care professionals will have the technical affinity to operate the AI-CDSS, potentially limiting the acceptance of the system [17]. Acceptance of an AI-CDSS is closely connected to explainability, accountability, and trust [39]. Increasing transparency of AI-systems leads to more trust in these systems and thus increases their acceptance [32]. If medical professionals cannot fully understand the recommendation of an AI-CDSS, it might result in conflicting situations [40]. This becomes evident considering the current legislation stating that even when using an AI-CDSS, medical professionals are responsible for the decisions [41]. However, this might change in the future with a CDSS improving and potentially even outperforming health care professionals [42].

In addition, professional caregivers need to be able to explain the use and functionality of the AI-CDSS to patients and their relatives [40,43,44]. In this context, the concept of digital divide seems important, which describes a decreased ability of older adults to use modern technologies compared with their younger counterparts [45]. However, a recent study could not detect interviewed geriatric patents’ reservation toward the use of smart sensors [46].

### Ethical Challenges of an AI-CDSS

Regarding the 4 core medico-ethical principles [19], the protection of patient autonomy seemed the most important, which reflects the current consent in medical practice about shared decision-making between patients and clinical professionals [47]. Although an AI-CDSS could support this, the identification of a patient’s values, beliefs, and aims is a prerequisite [48-50]. Overreliance on the AI-CDSS might lead to premature and rigid categorization of the patients according to preprogrammed categories, which are not sublime enough to catch intrinsic differences in a patient’s individual situation. Interviewees emphasized that all recommendations of an AI-CDSS must be reviewed critically by medical professionals to protect the patient’s will and autonomy.

Another central ethical code was the importance of preserving the caregiver-patient relationship—an aspect related to the principle of beneficence [19]. Interviewees demanded that they would not want the AI-CDSS to prejudge a patient. Patient-caregiver relationships were considered important for the recovery and self-efficiency of patients because of associated emotions and their trust-building effect [13]. Research about placebo effects also supports this position [51]. Fear of losing their professional identity was mentioned by some interviewees [52]. The best way to maintain professional identity and a trustful patient-caregiver relationships was seen in an open communication with the patient about the use of a CDSS, followed by informed consent.

Albeit only mentioned by a few participants, data protection and scientific evaluation of a new AI-CDSS are essential regarding the medico-ethical concept of nonmaleficence [53,54]. Especially, data protection seems to be crucial from an ethical perspective to protect sensitive personal patient data from theft, manipulation, or access by third parties [49,55]. In case of geriatric patients, who are often overwhelmed by modern processes of data gathering, storage, and processing, special responsibility falls on the developers of AI-CDSS and clinical professionals collecting the data.

A last aspect of medico-ethical reflection is justice, which was superficially touched upon only during the interviews. It has been argued that developing and maintaining a CDSS is cost-intensive and therefore could be afforded only in high-resource countries with a well-functioning health system and in big hospitals, such as university hospitals [13,56].

### Limitations

One limitation of the study was the rather low number of experts and an unproportionate distribution of health care professionals, with a higher representation of physicians. This does not allow for generalization of the results in a wider perspective. However, 15 interviewees should allow to capture about 85% (17/20) of possible topics, a phenomenon called “thematic saturation” [57], and in a smaller sample of interviewees, it is possible to focus on individual perspectives.

In addition, our information sheet did not explain the term AI to the interviewed health care professionals, so the reflections might have depended a lot on the individual knowledge. We, however, did not want to anticipate too much to broaden perspectives. Finally, the unique clinical information system, the status of hospital readiness for IT, and overall clinical structures could make it difficult to compare the findings with those from other hospitals, both nationally and internationally.

### Conclusions

An AI-CDSS was mostly considered to be beneficial, especially by providing health care professionals with an easily accessible data platform focused on geriatric needs and supporting geriatric comanagement and COC decisions. The most common concerns focused on maintaining the patient’s autonomy and preserving the patient-caregiver relationship, as well as smooth integration into existing workflows without extra tasks. Therefore, careful consideration must mainly be given to address technical demands (intuitive usability and easy access for all caregivers involved) and ethical challenges (maintaining the patient’s autonomy, explainability, and supportive character) during the development and implementation of a new AI-CDSS for geriatric patients in surgical departments.

## Supplementary material

10.2196/57899 Multimedia Appendix 1 Selected quotes for each main category.

## Abbreviations

AI: artificial intelligence
AI-CDSS: artificial intelligence–enhanced clinical decision support system
CDSS: clinical decision support system
COC: continuity of care
COREQ: Consolidated Criteria for Reporting Qualitative Research
EHR: electronic health record
HIS: hospital information system
