# Supplementary material for: Expectations and Requirements of Surgical Staff for an AI-Supported Clinical Decision Support System for Older Patients: Qualitative Study
Source: JMIR Aging. 2024 Dec 17;7:e57899. doi: 10.2196/57899 (PMC11683657; doi:10.2196/57899)
Supplement: Multimedia Appendix 1 [file aging-v7-e57899-s001.docx]

| **No** | **Quote** | **Healthcare professional (ID, years of affiliation with the department)** |
| --- | --- | --- |
| **Geriatric patient care** | | |
| 1 | *“So simultaneously we try to organize geriatrics, respite care, and look, if it is possible to organize home care. These are tasks, which we get. There is one task, but five other tasks have to be solved to actually discharge the patient.“* | Social service member (1, 8) |
| 2 | *“…we also often have difficulties about a geriatric rehabilitation afterwards. So there exist actually various forms, and somehow, we haven’t still completely understood it yet, I feel. Who needs a geriatric rehabilitation, who should get a complex geriatric treatment?“* | Assistant physician (3, 1) |
| 3 | *“Because of the daily constraints of occupancy, OP, discharge, and so on, we do not certainly find time, to deal more intensively with geriatric patients.”* | Senior physician (15, 13) |
| 4 | *“And…the mean length of stay is also very important for us. Money must be right in the hospital.“* | Social service member (1, 8) |
| 5 | *“…and we work here technically equipped with e-mail and fax and telephone.”* | Social service member (2, 2) |
| 6 | *“…to do it directly digitally, that we can see. Not again on paper and we have to run and look, where is the information attached to? So, this digital thing, we are missing a lot, really.”* | Social service member (1, 8) |
| 7 | *“…better communication, would be useful sometime…So, a better communication with family doctors, with nursing homes.“* | Senior physician (11, 2) |
| 8 | *“We clearly miss a lot information… Many preliminary information, telephone numbers from relatives… are often missing… If this information, if we could already do anamnesis here, at registration of patients. And access this information, it would be an incredible time saver.”* | Social service member (2, 2) |
| **Use of an AI-CDSS in geriatric care** | | |
| 9 | *“… it would actually make sense, if one could feed in all the data that we collect. Laboratory values, pre-existing illnesses, medication, information about relatives and from the social service, to see that at one glance.”* | Assistant physician (12, 6) |
| 10 | *“And should perhaps contain the drug interaction in a way, that it does not only say, does not go together, but immediately offers constructive solutions, how one could administer the medication, or something like that.”* | Senior physician (15, 13) |
| 11 | *“…chance for all, who are not regularly involved or deal with elderly people only occasionally… here arrive many, fell in nursing home, head laceration with Xarelto, get their CT- scan, get their suture, go home again. That is only very superficial…So there is definitely the chance, that you get a little more feeling for it or to see the patient maybe a little more holistic, then only bleeding head laceration.”* | Senior physician (11, 2) |
| 12 | *“Chances…because machine will always be better than men…the more data it is fed with, the better it becomes.”* | Senior physician (14, 9) |
| 13 | *“Well, I think the biggest factor is time here. Time expenditure should be as small as possible.“* | Senior physician (11, 2) |
| 14 | *„And then everybody, who is crucially involved, should have access. So, it is obviously also important, that information does not stick only to one person. But that all profession groups, also nurses, but also the social service, can directly access to the same version.”* | Senior physician (5, 19) |
| 15 | *“…it should be 100% compatible with the existing HIS, it should not be a big additional burden, has to pull out all the data from it of course, must not require any double entries.”* | Senior physician (15, 13) |
| 16 | *„It should be very up to date, it cannot be maintained once in 24 hours or so. It must be maintained very often.“* | Senior physician (7, 1) |
| 17 | *“But also for example, if somewhere there is a free bed in a geriatric hospital. If we would have something like that, that it is immediately shown there. That one can immediately use it and not one day after or so.”* | Senior physician (7, 1) |
| 18 | *“The handling, I think. I think that is the biggest problem, because not all people here are so firm in handling a computer and I think that the handling might be the problem later.”* | Physiotherapist (6, 22) |
| 19 | *“So, it is also an effort, let me say, to type it and so on and to enter all the data. I don’t think that this can be handled here, because if a lot is going on, one is happy when one… can accomplish the work and then on top to operate this program...”* | Senior physician (11, 2) |
| 20 | *“Problem might be, that it is difficult for someone to start new medication or to intervene. Because one already sees, what can go wrong in theory”* | Senior physician (14, 9) |
| **Ethical aspects of using an AI-CDSS in geriatric care** | | |
| 21 | *“…limits certainly are those, especially with geriatric patients, that they are extremely individual. So we have 85-year-olds who look like they are 60, and we have 60-year-olds who look like they are 80 and are biologically also like that.”* | Assistant physician (3,1) |
| 22 | *“…whether the decisions that are made, in their entire spectrum…are always ethically justifiable, naturally also depends on how many mistakes there are. So, objectifiable errors in such a decision. Of course, you have to check in a study whether this is okay.”* | Senior physician (7,1) |
| 23 | *“I don’t see any major problems with that. I mean it’s always like that, everything you do requires consent from the patient or legal guardian… But I personally don’t have any major concerns about the fact that all the information is now compiled in a dashboard and then used for optimal care.”* | Senior physician (5, 19) |
| 24 | *“…who is responsible for the decision…Is it the treating doctor, the one who makes the program. So, that will probably be ethical-legal…And it should be sufficiently validated… that one can really use it for decision finding…”* | Senior physician (15, 13) |
| 25 | *“Perhaps by speaking openly with the patient, that it exists and that one wants to use it. And by explaining exactly, for what it is used and what it should secure. And that it makes it only, if the patient agrees. So that one has a medical briefing about it and explains to the patient exactly, what it is and what it is for.”* | Assistant physician (12, 6) |
| 26 | *“…the patient should not know that I confer with the computer about what I do with him. So, I think, especially with older patients… they don’t understand something like that. Or they do not know artificial intelligence and if one tells them: Oh I have a computer program here and that told me you should go into a nursing home…I don’t think patients have a benefit if they know about it.”* | Senior physician (11, 2) |
